# Evaluation of apical extrusion of debris and centering ability in different nickel-titanium files during curved root canal preparation

**DOI:** 10.1186/s12903-023-03070-3

**Published:** 2023-06-15

**Authors:** Dongsheng Yu, Li Guo, Jing Gao, Jie Liu, Deqin Yang

**Affiliations:** 1 Northern Department of Endodontics, Stomatological Hospital of Chongqing Medical University, Chongqing, 404100 China; 2 Chongqing Key Laboratory of Oral Diseases and Biomedical Sciences, Chongqing, 404100 China; 3 Chongqing Municipal Key Laboratory of Oral Biomedical Engineering of Higher Education, Chongqing, 404100 China

**Keywords:** Apical extrusion of debris, Canal transportation, Centering ratio, Nickel-titanium file, Root canal preparation

## Abstract

**Background:**

Curved root canals lead to difficulties in cleaning, shaping and filling the root canal system. Apical extrusion of debris and root canal transportation are important factors causing postoperative complications. In clinical practice, commonly selected instruments include multifile NiTi systems, such as M3-Pro PLUS (M3-PRO), Orodeka Plex 2.0 (ODP), Rotate (ROT), and Protaper Gold (PTG), as well as single-file NiTi systems, such as M3-L Platinum 2019 (M3L), Waveone Gold (WOG), and Reciproc Blue (RCB). This study aimed to comprehensively evaluate the differences in the apical extrusion of debris and centering ability of the above NiTi files.

**Methods:**

Seventy 3D-printed resin teeth were used (n = 10). The apically extruded debris was collected in a preweighed centrifuge tube. The resin teeth with or without root canal preparation were cut into separate cross sections at 1 mm, 3 mm, 5 mm, and 7 mm away from the root apex, and then the root canal transportation and centering ratio of each cross section were calculated.

**Results:**

Apical extrusion of debris was highest in RCB but lowest in OD-P (P < 0.05). Root call deviation was lowest in ROT at the 3 mm level, in PTG at the 5 mm level, and in PTG and ROT at the 7 mm level (P < 0.05). The centering ratio of NiTi files was highest in the RCB group at the 3 mm level, in the PTG group at the 5 mm level, in the ROT group at the 7 mm level (P < 0.05).

**Conclusions:**

For NiTi files with the same system, the cross-sectional design is the greatest factor affecting the extrusion of debris, and motion mode is the second. In addition, the multifile system could reduce the degree of root canal transportation.

**Supplementary Information:**

The online version contains supplementary material available at 10.1186/s12903-023-03070-3.

## Background

Root canal therapy is an effective method for treating pulp and periapical diseases [1]. Since 1853, the key to root canal therapy has been eliminating the source of infection [2]. Root canal preparation and disinfection are the main methods used to remove the infection source entirely [3]. Root canal preparation aims to clear the diseased pulp tissue and bacterial products in the root canal while providing sufficient space to deliver drugs, dressings, and other materials into the canal. During root canal preparation, the mechanical action of preparation instruments and the chemical effects of irrigation fluid play a crucial role in achieving root canal debridement and disinfecting the root canal system [4]. During preparation, dentine debris, pulp tissue residue, bacteria and their products may be pushed to the end of the root canal or out of the apical foramen into the periapical tissue [5]. This is among the primary causes of postoperative pain, such as flare-ups after root canal therapy [6]. In addition, due to the complex anatomy of the root canal and some limitations of preparation instruments, adverse conditions, such as step formation, lateral perforation and root canal transportation, may occur during root canal preparation [7]; as a result, the long-term curative effect and even the preservation of natural teeth are affected.

The rate of curved root canals is very high, and approximately 84% of root canals are curved [8]. Curved root canals lead to difficulties in cleaning, shaping and filling the root canal system. Compared to straight root canals, the preparation of curved root canals necessitates greater instrument performance. To improve the success rate of root canal treatment in curved root canals, all aspects of preparation instruments, such as the material, manufacturing techniques, and morphological structure, are continually being developed and improved. In 2008, a single-file for reciprocating motion was introduced. In the single-file system, several problems are eliminated, including the need to frequently replace instruments and constantly adjust motor parameters in multifile systems, further improving the efficiency of root canal preparation [9]. In this study, representative instruments of multifile NiTi systems, such as M3-Pro PLUS (M3-PRO), Orodeka Plex 2.0 (OD-P), Rotate (ROT), and Protaper Gold (PTG), as well as single-file NiTi systems, such as M3-L Platinum 2019 (M3L), Waveone Gold (WOG), and Reciproc Blue (RCB), were selected as the experimental objects. In research on the amount of apical extruded debris, only Elashiry et al. [10] reported that RCB produced more debris than that of WOG. In addition, although some researchers studied apical debris extrusion of ROT [11], there was no comparison with any NiTi files, such as M3L, M3-PRO, and so on. Regarding the centering ability, only Silva et al. [12] reported that the root canal transportation of RCB is smaller than that of PTG at the apical third. In summary, no studies have comprehensively evaluated the differences in the apical debris extrusion and centering ability of the seven NiTi files.

In investigations on the performance of NiTi instruments, the isolated teeth were mostly used as research models. Due to individual differences, it is difficult to standardize the morphology, apical diameter and tooth hardness of natural teeth; hence, the sample consistency is low, and the reference value of the experimental results is limited. However, the consistency of 3D-printed teeth is excellent, as almost the same working length, root canal curvature and root canal morphology are achieved, which can effectively eliminate experimental errors. According to Ronald [13], the hardness of resin models was not significantly different from that of dentin, and there were no problems, such as resin softening or instrument separation, during the preparation of the resin model. Gok, Yoshio and many other scholars [14–16] have indicated that 3D-printed teeth can gradually replace isolated teeth and serve as new models for evaluating the performance of root canal preparation instruments.

This study used 3D-printed resin teeth as samples to systematically compare apical debris extrusion and centering ability among M3L, WOG, RCB, M3-PRO, OD-P, ROT, and PTG. This is expected to provide a reference and experimental basis for the preferred selection of preparation instruments in root canal therapy, especially in curved root canal therapy.

## Methods

### Selection and calculation of samples

The sample size was estimated by analysis of variance (GPower 3.1.9 software) based on data from previous studies [17]. Considering the effect size of 0.5, the test power of 0.85, α = 0.05 and 7 experimental groups, the sample size of each group was set to at least 10 teeth after calculation. A total of 70 3D-printed resin teeth imitating the right maxillary canine were used in this study, and the shape of the resin tooth is shown in Figure S1. According to the method proposed by Schneider [18], the root canal curvature of resin teeth was measured to be 45°. To standardize the operation process and unify the reference point, the crown of each resin tooth was removed 2 mm from the tip using a dental high-speed handpiece, and the final length of all teeth was uniform at 19 mm. The access cavity of the resin teeth were then opened by a high-speed split drill and ball drill with an air-water spray cooling system. The canal patency was verified by inserting a #10 K file (Dentsply, Switzerland) until the tip of the file could be seen in the apical foramen under a dental stereomicroscope (Zeiss, Germany). Then, the length of the file was recorded, and the working length was the length of the file minus 1 mm.

### Experimental group design

Seventy 3D-printed resin teeth were divided into 7 experimental groups (n = 10). Group 1: M3L Platinum Edition 2019 (M3L, United Dental Changzhou, China); Group 2: M3-Pro Plus (M3-Pro, United Dental Changzhou, China); Group 3: Orodeka Plex 2.0 (OD-P, Orodeka, China); Group 4: Reciproc Blue (RCB, VDW, Germany); Group 5: Rotate (ROT, VDW, Germany); Group 6: Waveone Gold (WOG, Dentsply, Switzerland); and Group 7: Protaper Gold (PTG, Dentsply, Switzerland). The alloy type, movement mode, cross-sectional shape and system composition of aforementioned NiTi files are shown in Table S1.

### Root canal preparation

All root canal preparations were performed by one of the authors. All NiTi instruments were equipped with an X-Smart Plus motor and matched handle. Distilled water served as the root canal irrigation solution. A new set of corresponding NiTi files was used individually for each resin tooth. The root canal was prepared by “pecking” or “pulling”, advancing approximately 2–3 mm each time until the apical diameter was 0.25 mm. The file was immediately withdrawn from the root canal when the movement of the file was resisted, and the file rotation time in the root canal did not exceed 5 s. The surface of the file was cleaned by a 75% alcohol cotton ball after the file was withdrawn or replaced, and the root canal was rinsed with 2 ml distilled water, back-filtered using #10 K, and rinsed with 1 ml distilled water again. The above cycle was repeated until the preparation was completed. The motion parameters of the instruments are shown in Table S2. In addition, multifile systems were used from top to bottom.

### Collection and weighing model for apically extruded debris

The model for the collection of apically extruded debris was first established by Myers and Montgomery [19]. In this study, we modified the model based on the latest relevant articles [20, 21]. Figure S2 shows the simplified process of this experiment. The details are as follows: (1) A 20 ml centrifuge tube without the cap was weighed on an electronic balance (accuracy: 10^− 4^ g). Each tube was measured three times, and the average value was recorded as the initial weight of the centrifuge tube. (2) A hole was punched in the center of the centrifuge tube lid. The resin teeth were fixed on the centrifuge tube lid with the cemento-enamel junction as the boundary, and a 27-gauge needle was inserted to balance the pressure. The gap was sealed with light-curing resin. The centrifuge tube was fixed on the glass bottle to facilitate the operator’s operation and prevent the operator from touching the outer wall of the tube. A rubber dam was used to isolate the tooth from the underlying device and prevent the operator from observing the apical foramen during preparation. The experimental device is shown in Figure S3. (3) The total amount of irrigant was 10 ml for either single or multiple files, and root canal was rinsed with 2 ml distilled water every time the single file was removed or multiple files were replaced. If the amount of irrigant was less than 10 ml after preparation, we continued rinsing until 10 ml of irrigant was available. Then, the root apex was rinsed in the tube with 1 ml distilled water to collect debris adhering to the apical surface. In other words, 11 ml distilled water was used in the process. (4) Centrifuge tubes were placed in a 70 ℃ incubator for 2 weeks until completely dry and then weighed. The average value was the final weight of the tube. The weight of the apically extruded debris is the final weight of the tube minus the initial weight of the tube.

### Centering ability

The centering ability of each file was determined by calculating the root canal transportation and centering ratio at different observation planes. Based on previous studies [22], planes at 1 mm, 3 mm, 5 and 7 mm from the apical foramen were used as the observation planes in this study, and the resin teeth after root canal preparation were cut into separate cross sections at the aforementioned four positions (Figure S4). Three resin teeth without root canal preparation were also cut into separate cross sections at the same position, and their average value was used as the data before root canal preparation. According to the method proposed by Gambill [23] (Figure S5), each cross section was observed under a stereo microscope (Zeiss, Germany), and the obtained images were measured using ZEN 2011 software (Figure S6). The following data of each observation plane were recorded: A1: The minimum distance between the mesial root surface and the mesial root canal wall before root canal preparation; A2: The minimum distance between the mesial root surface and the mesial root canal wall after root canal preparation; B1: The minimum distance between the distal root surface and the distal root canal wall before root canal preparation; B2: The minimum distance between the distal root surface and the distal root canal wall after root canal preparation. Root canal transportation = [(A1-A2) - (B1-B2)]. The closer the value is to 0, the smaller the root canal transportation; a positive value represents mesial movement, while a negative value represents distal movement. The plus or minus signs only indicate the direction of root canal transportation and have no mathematical significance. When (A1-A2) is less than (B1-B2), the axis center rate = (A1-A2)/(B1-B2); when (A1-A2) is greater than (B1-B2), the axis center rate = (B1-B2)/(A1-A2). The closer the centering ratio is to 1, the better the centering ability.

### Statistical analysis

SPSS 26.0 software was used for statistical analysis. Levene’s test was first used to verify the homogeneity of variance of the data. Data with homogeneous variance (*P*>0.05) were then statistically analyzed using one-way ANOVA analysis and post hoc Tukey’s test with the significance level set at 5%. Otherwise, the nonparametric Kruskal‒Wallis test was applied for the data without homogeneous variance (*P < 0.05*). If there was a certain difference between groups, the Bonferroni correction method was further used for pairwise comparison with the significance level set at 5%.

## Results

### Amount of apically extruded debris

**Fig. 1:**
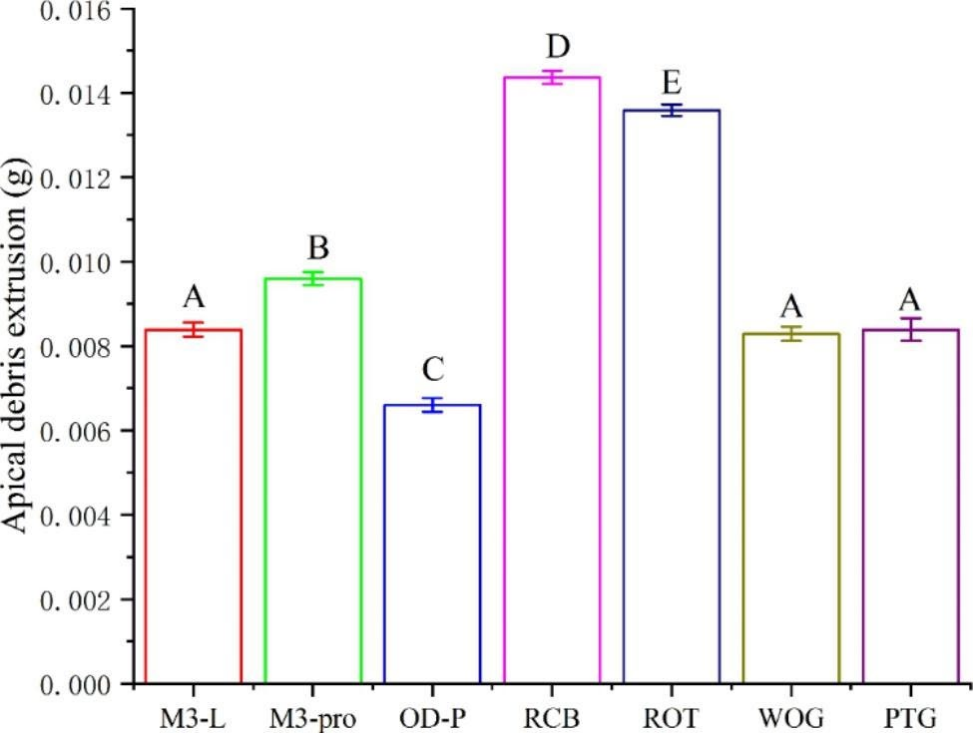
The apical debris extrusion in each group. Groups marked by different letters are significantly different from each other (*P* < 0.05), whereas marked with the same letter are not (*P* > 0.05)

The data were confirmed to meet homogeneity variance by Levene’s test (*P* > 0.05), so one-way ANOVA analysis and post hoc Tukey’s test were used for further analysis. The amount of apically extruded debris was ranked as follows: RCB > ROT > M3-PRO > M3L, WOG, PTG > OD-P. The amount of apically extruded debris was highest in the RCB group, and lowest in the OD-P group (*P < 0.05*). There was no significant difference among the M3L, WOG and PTG groups (*P* > 0.05); in addition, the other groups all showed significant differences between each other (*P < 0.05*) (Table 1; Fig. 1).

**Table 1:** Mean, standard deviation, and *F*-test results of the amount of apically extruded debris in each experimental group

Groups	Mean (g)	Standard deviation	*F*-test	
			F	P
M3-L platinum 2019	0.008390^ A^	0.0001663	2719.079	0.000
M3-PRO plus	0.009600^B^	0.0001563		
Orodeka Plex 2.0	0.006600^ C^	0.0001633		
Reciproc blue	0.014370^D^	0.0001567		
Rotate	0.013593^E^	0.0001376		
Waveone Gold	0.008290^ A^	0.0001663		
Protaper Gold	0.008390^ A^	0.0002685		

There are significant differences between groups marked by different superscript letters (*P < 0.05*). There is no significant difference between groups marked with the same superscript letter (*P* > 0.05).

### Centering ability

#### Root canal transportation of the observation plane at 1 mm from the root apex

**Fig. 2:**
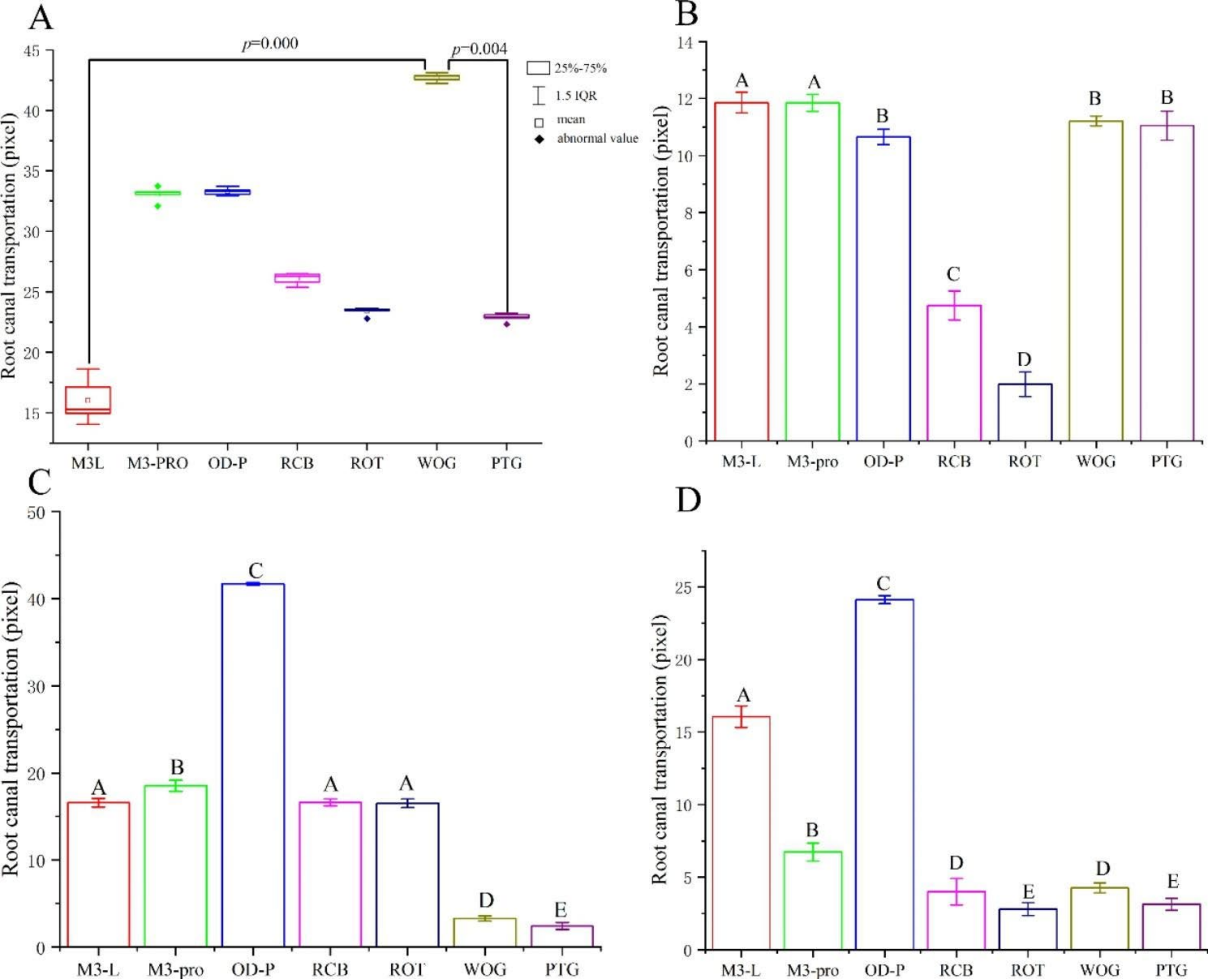
Root canal transportation of four observation planes. (**A**) Root canal transportation of plane at 1 mm from the root apex. (**B**) Root canal transportation of the plane at 3 mm from the root apex in each group. (**C**) Root canal transportation of the plane at 5 mm from the root apex in each group. (**D**) Root canal transportation of the plane at 7 mm from the root apex in each group. Groups marked by different letters are significantly different from each other (*P* < 0.05), whereas marked with the same letter are not significantly different (*P* > 0.05). Abbreviations: IQR, interquartile range; M3-L, M3-L Platinum 2019; M3-PRO, M3-Pro PLUS; OD-P, Orodeka Plex 2.0; RCB, Reciproc Blue; ROT, Rotate; WOG, Waveone Gold; PTG, Protaper Gold

Levine’s test showed that the root canal transportation of the plane at 1 mm from the root apex did not meet the homogeneity of variance (*P* = 0.000, *P < 0.05*). The nonparametric Kruskal‒Wallis test indicated that there were significant differences in root canal transportation among the different groups (H = 32.189, *P* < 0.000). After Bonferroni correction was performed, the results showed that there was a significant difference between M3L and WOG (*P* = 0.000) and between WOG and PTG (*P* = 0.004). In addition, there was no significant difference among the remaining groups (Fig. 2A).

#### Root canal transportation of the observation plane at 3 mm from the root apex

Levine’s test confirmed that root canal transportation of the plane at 3 mm from the root apex met the homogeneity of variance (*P* = 0.456, *P* > 0.05); furthermore, one-way ANOVA analysis and post hoc Tukey’s test were used (α = 0.05). The root call deviation data were ranked as follows: M3L, M3-PRO > WOG, PTG, OD-P > RCB > ROT. The root call deviation was highest in M3L and M3-PRO but lowest in ROT. There was no significant difference between M3L and M3-PRO, among WOG, PTG, and OD-P (*P* > 0.05), and the differences among the other groups were statistically significant (*P < 0.05*) (Table 2; Fig. 2B).

**Table 2:** Mean, standard deviation, and *F*-test results of root canal transportation of the observation plane at 3 mm from the root apex in each experimental group

Groups	Mean (pixel)	Standard deviation	*F*-test	
			F	P
M3-L platinum 2019	-11.85600^ A^	0.359529	542.354	0.000
M3-PRO plus	-11.84600^ A^	0.297751		
Orodeka Plex 2.0	10.65320^B^	0.268996		
Reciproc blue	-4.74980^ C^	0.509213		
Rotate	-1.98760^D^	0.428302		
Waveone Gold	-11.21360^B^	0.168897		
Protaper Gold	-11.04900^B^	0.513371		

The positive value represents mesial movement, while the negative value means distal movement. The plus or minus signs only indicate the direction of root canal transportation and have no any mathematical significance. There are significant differences between groups marked by different superscript letters (*P < 0.05*). There is no significant difference between groups marked with the same superscript letter (*P* > 0.05).

#### Root canal transportation of the observation plane at 5 mm from the root apex

The root canal transportation of the plane at 5 mm from the root apex was verified to meet homogeneity of variance using Levine’s test (*P* = 0.063, *P* > 0.05). The root canal transportation was ordered as follows: OD-P > M3-PRO > M3L, RCB, ROT > WOG > PTG. The root call deviation was notably highest in OD-P but lowest in PTG. There was no significant difference among the M3L, RCB and ROT groups (*P* > 0.05); beyond that, the differences among the remaining groups were statistically significant (*P < 0.05*) (Table 3; Fig. 2C).

**Table 3:** Mean, standard deviation, and *F*-test results of root canal transportation of the observation plane at 5 mm from the root apex in each experimental group

Groups	Mean (pixel)	Standard deviation	*F*-test	
			F	P
M3-L platinum 2019	16.58300^ A^	0.494313	4418.126	0.000
M3-PRO plus	18.53340^B^	0.637081		
Orodeka Plex 2.0	41.70580^ C^	0.144895		
Reciproc blue	16.62460 ^A^	0.386281		
Rotate	16.52880^ A^	0.501947		
Waveone Gold	3.29980 ^D^	0.295201		
Protaper Gold	-2.43640 ^E^	0.419523		

The positive value represents the mesial movement, while the negative value means the distal movement. The plus or minus signs only indicate the direction of root canal transportation without any mathematical significance. There are significant differences between groups marked by different superscript letters (*P < 0.05*). There is no significant difference between groups marked with the same superscript letter (*P* > 0.05).

#### Root canal transportation of the observation plane at 7 mm from the root apex

Levene’s test showed that the root canal transportation of the plane at 7 mm from the root apex satisfied the homogeneity of variance (*P* = 0.113, *P* > 0.05). The root canal transportation was ranked in the following order: OD-P > M3L > M3-PRO > WOG, RCB > PTG, ROT. The root canal transportation was clearly highest in the OD-P group, and lowest in the PTG and ROT groups. In addition, there was no significant difference between WOG and RCB, PTG and ROT (*P* > 0.05), and the differences among the other groups were statistically significant (*P < 0.05*) (Table 4; Fig. 2D).

**Table 4:** Mean, standard deviation, and *F*-test results of root canal transportation of the observation plane at 7 mm from the root apex in each experimental group

Groups	Mean (pixel)	Standard deviation	*F*-test	
			F	P
M3-L platinum 2019	16.05600^ A^	0.738583	1014.762	0.000
M3-PRO plus	-6.73240 ^B^	0.617122		
Orodeka Plex 2.0	24.12060^ C^	0.273250		
Reciproc blue	-4.00600^D^	0.908378		
Rotate	-2.79900 ^E^	0.442430		
Waveone Gold	4.26460^D^	0.356044		
Protaper Gold	3.13380 ^E^	0.411802		

The positive value represents the mesial movement, while the negative value means the distal movement. The plus or minus signs only indicate the direction of root canal transportation without any mathematical significance. There are significant differences between groups marked by different superscript letters (*P* < 0.05). There is no significant difference between groups marked with the same superscript letter (*P* > 0.05).

### Centering ratio

#### Centering ratio of the plane at 1 mm from the root apex

**Fig. 3:**
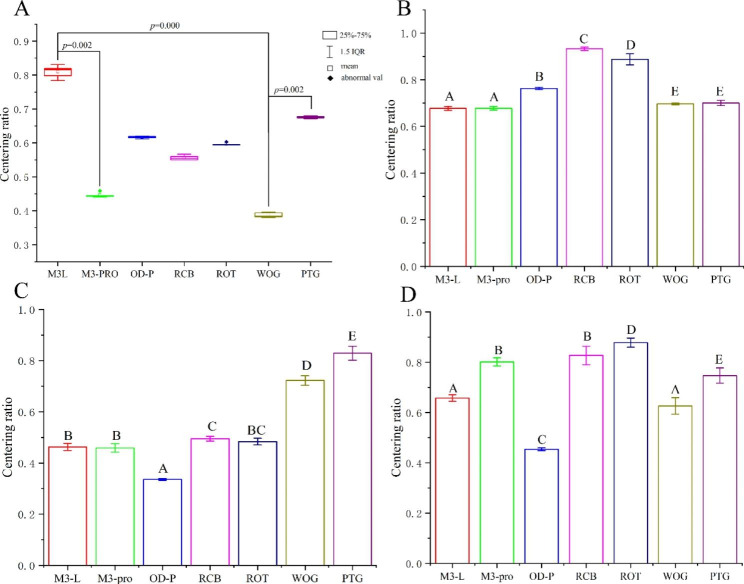
Centering ratio of four observation planes. (**A**) Centering ratio of the plane at 1 mm from the root apex in each group. (**B**) Centering ratio of the plane at 3 mm from the root apex in each group. (**C**) Centering ratio of the plane at 5 mm from the root apex in each group. (**D**) Centering ratio of the plane at 7 mm from the root apex in each group. Groups marked by different letters are significantly different from each other (*P* < 0.05), whereas marked with the same letter are not significantly different (*P* > 0.05). Abbreviations: IQR, interquartile range; M3-L, M3-L Platinum 2019; M3-PRO, M3-Pro PLUS; OD-P, Orodeka Plex 2.0; RCB, Reciproc Blue; ROT, Rotate; WOG, Waveone Gold; PTG, Protaper Gold

Levene’s test showed that the centering ratio of the plane at 1 mm from the root apex did not satisfy the homogeneity of variance (*P* = 0.001, *P* < 0.05). The nonparametric Kruskal‒Wallis test indicated that there were significant differences in the centering ratio between different groups (H = 33.347, *P* = 0.000). The Bonferroni correction further showed that there was significant differences between M3L group and WOG group, M3L group and M3-PRO group, and WOG group and PTG group (*P* < 0.05); beyond that, there was no significant difference among the other groups (Fig. 3A).

#### Centering ratio of the plane at 3 mm from the root apex

The centering ratio of the plane at 3 mm from the root apex satisfied the homogeneity of variance by Levene’s test (*P* = 0.146, *P* > 0.05). Therefore, one-way ANOVA analysis and post hoc Tukey’s test were used for further analysis (α = 0.05). The center rate was ranked as follows: RCB > ROT > OD-P > WOG, PTG > M3L, M3-PRO. The center rate was highest in the RCB group, and lowest in the M3L and M3-PRO groups. In addition, there was no significant difference between WOG and PTG, M3L and M3-PRO (*P* > 0.05), and the differences between the other groups were statistically significant (*P* < 0.05) (Table 5; Fig. 3B).

**Table 5:** Mean, standard deviation, and *F*-test results of centering ratio of the observation plane at 3 mm from the root apex in each experimental group

Groups	Mean	Standard deviation	*F*-test	
			F	P
M3-L platinum 2019	0.67740^ A^	0.008620	427.431	0.000
M3-PRO plus	0.67760 ^A^	0.008142		
Orodeka Plex 2.0	0.76260 ^B^	0.004722		
Reciproc blue	0.93280 ^C^	0.007190		
Rotate	0.88760 ^D^	0.023586		
Waveone Gold	0.69640 ^E^	0.003507		
Protaper Gold	0.70060 ^E^	0.011632		

There are significant differences between groups marked by different superscript letters (*P* < 0.05). There is no significant difference between groups marked with the same superscript letter (*P* > 0.05).

#### Centering ratio of the plane at 5 mm from the root apex

The center rate of the plane at 5 mm from the root apex had homogeneity of variance by Levene’s test (*P* = 0.180, *P* > 0.05). The centering ratio was ordered as follows: PTG > WOG > RCB, ROT > ROT, M3L, M3-PRO > OD-P. The center rate was obviously highest in the PTG group, whereas lowest in the OD-P group. There was no significant difference between RCB and ROT, among M3L, M3-PRO and ROT (*P* > 0.05), and the differences between the other groups were statistically significant (*P* < 0.05) (Table 6; Fig. 3C).

**Table 6:** Mean, standard deviation, and *F*-test results of centering ratio of the observation plane at 5 mm from the root apex in each experimental group

Groups	Mean	Standard deviation	*F*-test	
			F	P
M3-L platinum 2019	0.46280^B^	0.013900	559.117	0.000
M3-PRO plus	0.45860 ^B^	0.016577		
Orodeka Plex 2.0	0.33560 ^A^	0.003715		
Reciproc blue	0.49460 ^C^	0.009423		
Rotate	0.48360 ^BC^	0.013069		
Waveone Gold	0.72200 ^D^	0.018695		
Protaper Gold	0.82880 ^E^	0.027326		

There are significant differences between groups marked by different superscript letters (*P* < 0.05). There is no significant difference between groups marked with the same superscript letter (*p* > 0.05).

#### Centering ratio of the plane at 7 mm from the root apex

The center rate of the plane at 7 mm from the root apex was confirmed to meet the homogeneity of variance using Levene’s test (*P* = 0.117, *P* > 0.05). The centering ratio was ranked as follows: ROT > RCB, M3-PRO > PTG > M3L, WOG > OD-P. The centering ratio was highest in the ROT group (the highest average value closest to 1), and clearly lowest in the OD-P group. There was no significant difference between RCB and M3-PRO, M3L and WOG (*P* > 0.05), and the differences between the other groups were statistically significant (*P* < 0.05) (Table 7; Fig. 3D).

**Table 7:** Mean, standard deviation, and *F*-test results of centering ratio of the observation plane at 7 mm from the root apex in each experimental group

Groups	Mean	Standard deviation	*F*-test	
			F	P
M3-L platinum 2019	0.65780^ A^	0.013103	178.904	0.000
M3-PRO plus	0.80140 ^B^	0.016149		
Orodeka Plex 2.0	0.45420 ^C^	0.005541		
Reciproc blue	0.82720 ^B^	0.036840		
Rotate	0.87820 ^D^	0.018102		
Waveone Gold	0.62680 ^A^	0.032798		
Protaper Gold	0.74720 ^E^	0.030161		

There are significant differences between groups marked by different superscript letters (*P* < 0.05). There is no significant difference between groups marked with the same superscript letter (*P* > 0.05).

## Discussion

It is well known that dentin debris, especially infected debris in the infected root canal can be extruded by Ni-Ti files, causing postoperative complications [5]. Hence, the representative instruments of multifile systems, such as M3-PRO, OD-P, ROT, PTG, and RCB, as well as the typical instruments of single-file systems, such as M3L, WOG, and RCB, were systematically compared in this study. We modified the experimental model for collecting the debris based on the model established by Myers and Montgomery [24]. The centrifuge tube for collection of debris was placed on a glass bottle, and a rubber dam was used to isolate the device below the cemento-enamel junction of the tooth, which is similar to the clinical situation. The principal advantage of this model is that the operator can only observe the root canal orifice, thus the experimental results are not affected by bservations of the apical foramen.

Elashiry [10] et al. compared the difference in the extrusion of apical debris between WOG, RCB, and HyFlex EDM, and found that the amount of apically extruded debris was statistically similar between RCB and WOG. Recai Zan [25] et al. found that there was no significant difference in the amount of apically extruded debris between the WOG and PTG groups in isolated mandibular incisors. In this study, we found that WOG and PTG were statistically similar, while the RCB value was higher, which may be because the length of resin teeth used in this study was longer than that of real teeth in other studies. According to a meta-analysis study, no significant difference was observed in the amount of apically extruded debris between a single-file system and multifile system [26]. Our results were consistent with this conclusion, as no significant differences were observed between the single-file system WOG and multifile system PTG. A recent meta-analysis [27] demonstrates that a single-file reciprocating system tends to cause greater debris extrusion than that of a single-file rotating system. Our results also revealed that the apically extruded debris was higher in the reciprocating system RCB than in the rotating system M3L. Unexpectedly, there was no clear difference in debris extrusion between reciprocating system WOG and rotating system M3L, which may be because the parallelogram-shaped cross section of WOG reduced the extrusion of apical debris. Namely, the effect of the cross-sectional shape even exceeds the effect of the movement mode on the extrusion of apical debris.

In the present study, the extrusion of apical debris was lowest in the OD-P group. This might be because the tip of OD-P, a safe guide tip without a blade, provides a guiding rather than cutting ability, reducing the extrusion of dentin debris from the apical part of the root canal. Moreover, the unique “3S”-shaped cross section and the gradually elongated thread design of OD-P increase the discharge space of debris during root canal preparation, which greatly lessens the extrusion of debris through the apical foremen. ROT is another new NiTi system from VDW and introduces an S-shaped cross section with higher cutting efficiency on the basis of RCB. This cross section decreases the cutting area of dentin and therefore the production of dentin debris, which may explain why the extrusion of apical debris is less in ROT than in RCB. These findings agree with the results of several studies, which indicates that the cross-sectional design of the instrument is the main parameter affecting the extrusion of apical debris [28–30]. In earlier studies, there was a significant difference between the hardness of the resin block and dentin, and the heat generated during instrument rotation may soften the resin block, thereby increasing the amount of debris. However, with the progress of material technology, the above shortcomings have been improved, and the hardness of resin models was not significantly different from that of dentin. In the research by Ronald [13] and this study, these shortcomings were not observed. However, the 3D-printed resin teeth used in this study could not fully mimic the physiological structures of dentin, so it is not possible to determine whether different instruments cause different dentin microcracks during root canal preparation.

In the present study, the centering ability of different NiTi files was evaluated by calculating the root canal transportation and centering ratio in different planes according to the method proposed by Gambill [23]. Resin teeth were cut into separate cross sections at 1 mm, 3 mm, 5 mm, and 7 mm from the root apex, and each cross section was observed and then measured. The results showed that the root canal transportation and centering ratio of the plane at 1 mm from the root apex did not satisfy the homogeneity of variance; moreover, there was no significant difference in most of the data. This may be because the area of the plane at 1 mm from the root tip is too small, leading to a sharp increase in mechanical error when cutting the cross section. According to the results of other level planes, we found that RCB and ROT exhibit relatively superior centering ability, which may be related to their special blue thermal processing. The centering ability of OD-P was second to that of RCB and ROT at 3 mm but was worst at 5 and 7 mm. The centering ability of M3L and M3-PRO was worst at 3 mm from the root tip and only better than OD-P at 5 mm, which may be closely connected with their material and processing technology. In addition, RCB showed less root canal transportation than that of PTG at 3 mm, which was consistent with the results of Silva et al. [12]. However, PTG displayed smaller root canal transportation than that of RCB at 5 and 7 mm, which may be associated with the variable taper design of PTG used for preparing the middle and cervical parts of the canal. For different file systems from the same brand, the multifile system PTG showed less root canal transportation and better centering ability than that of the single-file system WOG at 3 mm, 5 and 7 mm, although the difference was not significant at the 3 mm level. In addition, there was no significant difference in the amount of apical debris between PTG and WOG and no significant difference in the centering ability of the single-file system M3L and the multifile system M3-PRO at 3 and 5 mm. This may have occurred because M3L and M3-PRO share the same continuous rotational motion pattern. At the level of 7 mm, M3-PRO showed better centering ability than M3L, while M3-PRO simultaneously produced more apical debris than that of M3L. In addition, compared to single-file RCB, the multifile system ROT showed less canal transportation at every level, although the difference at 5 mm is not significant. However, RCB exhibited a higher axial center ratio than that of ROT at 3 mm level. Although ROT exhibited the second highest amount of apically extruded debris among the seven files, its apically extruded debris and centering ability were improved compared to RCB, which may provide a good reference for improving the NiTi file in the future.

Taken together, the results indicated that OD-P showed the least amount of apically extruded debris and relatively good centering ability in the apical part of the root canal; therefore, OD-P is most suitable for heavily infected root canals. Given that OD-P shows the worst centering ability in the middle and coronal parts of the root canal, other brands of opening files and unblocking files can be used synergistically to complete the upper-middle root canal preparation. RCB and ROT exhibit the best centering ability in this study and are suitable for preparing teeth with thin canal walls to prevent lateral perforation. PTG can be used in the upper-middle root canals to reduce the possibility of perforation during root canal preparation due to its minimal deviation. Although newly developed NiTi files, such as M3L and M3-PRO, still show a slight deficiency in centering ability compared with WOG and PTG, there is no significant difference in the apical extrusion of debris among them; hence, the clinical application prospects of M3L and M3-PRO are promising.

## Conclusion

For NiTi files with the same system, the cross-sectional design is the greatest factor affecting the apical extrusion of debris. Moreover, compared to the single-file reciprocating system, the single-file rotating system tended to produce more apical debris than single-file reciprocating system, but the effect of motion mode was inferior to the effect of the cross-sectional design on the extrusion of apical debris. In terms of centering ability, compared to a single-file system, multifile system can better reduce root canal transportation.

Specifically, OD-P caused the least apical extrusion of debris and can be used for heavily infected root canals. RCB or ROT can be used for thin canal preparation to avoid canal perforation as much as possible. Moreover, NiTi files from different brands can be mixed to compensate for the shortcomings of using single brand files, and distinct nickel-titanium files can be used for the upper-middle and apical parts of the root canal, respectively. All NiTi files exhibit advantages and disadvantages, but we can choose and combine the NiTi files flexibly to achieve the best therapeutic outcome in clinical practice.

## Electronic supplementary material

Below is the link to the electronic supplementary material.

**Supplementary Material 1**: **Table S1** Overview of seven new nickel-titanium files. **Table S2** Motion parameters of all NiTi files used in experiment. **Figure S1**. 3D printed resin teeth imitating the right maxillary canine. A: mesial surface, B: distal surface, C: labial surface, D: palatal surface. **Figure S2**. Schematic diagram for collection of apical debris. **Figure S3**. Schematic diagram of the apical debris collection model. **Figure S4**. Schematic diagram of cross sections at different distance from the root apex. **Figure S5**. Schematic diagram showing the root canal transportation and centering ratio measurement. The red area represents the root canal lumen before root canal preparation, while the blue area represents the root canal lumen after root canal preparation. **Figure S6**. Cross-sectional images before and after root canal preparation in different experimental groups.

## Data Availability

All data analyzed during this study are included in this article.

## Abbreviations

NiTi: Nickel titanium
M3-PRO: M3-Pro PLUS
OD-P: Orodeka Plex 2.0
ROT: Rotate
PTG: Protaper Gold
M3L: M3-L Platinum 2019
WOG: Waveone Gold
RCB: Reciproc Blue
