# Supplementary material for: Evaluation of apical extrusion of debris and centering ability in different nickel-titanium files during curved root canal preparation
Source: BMC Oral Health. 2023 Jun 15;23:395. doi: 10.1186/s12903-023-03070-3 (PMC10273756; doi:10.1186/s12903-023-03070-3)
Supplement: Supplementary file 1 — Supplementary Material 1: Table S1 Overview of seven new nickel-titanium files. Table S2 Motion parameters of all NiTi files used in experiment. Figure S1. 3D printed resin teeth imitating the right maxillary canine. A: mesial surface, B: distal surface, C: labial surface, D: palatal surface. Figure S2. Schematic diagram for collection of apical debris. Figure S3. Schematic diagram of the apical debris collection model. Figure S4. Schematic diagram of cross sections at different distance from the root apex. Figure S5. Schematic diagram showing the root canal transportation and centering ratio measurement. The red area represents the root canal lumen before root canal preparation, while the blue area represents the root canal lumen after root canal preparation. Figure S6. Cross-sectional images before and after root canal preparation in different experimental groups. [file 12903_2023_3070_MOESM1_ESM.docx]

**supplementary materials**

**Table S1-S2**

**Figure S1-S6**

Data statement: All supplementary materials provide a detailed explanation for the methods section.

**Table S1** Overview of seven new nickel-titanium files

| System type | Instrument name | Movement mode | Cross section shape | Alloy type |
| --- | --- | --- | --- | --- |
| Single-file | M3-L Platinum 2019 | continuous rotation | double S shape | L wire |
|  | Reciproc Blue | reciprocating motion | S-shape | CM blue wire |
|  | Waveone Gold | reciprocating motion | parallelogram | CM gold wire |
| Multi-file | M3-Pro PLUS | continuous rotation | S-shape | CM wire |
|  | orodeka plex 2.0 | continuous rotation | 3S-shape | CM wire |
|  | Rotate | continuous rotation | improved S-shape | CM blue wire |
|  | Protaper Gold | continuous rotation | convex triangle | CM gold wire |

**Table S2** Motion parameters of all NiTi files used in experiment.

| Instrument components | | Rotate speed (rpm) | Torque (Ncm) | |
| --- | --- | --- | --- | --- |
| Waveone Gold  Reciproc Blue  M3-L Platinum 2019 | | Set to " Waveone Gold " mode | | |
|  |  | Set to "Reciproc" mode | | |
|  |  | 400 | | 2.0 |
| M3-Pro PLUS | 17/12 file | 300 | | 3.0 |
|  | 12/05 file | 350 | | 1.5 |
|  | 16/06 file | 350 | | 1.5 |
|  | 18/05 file | 350 | | 1.5 |
|  | 25/06 file | 350 | | 1.5 |
| Orodeka plex 2.0 | 15/03 file | 300 | | 1.5 |
|  | 15/08 file | 500 | | 2.5 |
|  | 20/05 file | 500 | | 2.5 |
|  | 25/06 file | 500 | | 2.5 |
| Rotate | 15/04 file | 350 | | 2.1 |
|  | 20/05 file | 350 | | 2.3 |
|  | 25/06 file | 350 | | 2.3 |
| Protaper Gold | SX file | 300 | | 3.0 |
|  | S1 file | 300 | | 3.0 |
|  | S2 file | 250 | | 1.0 |
|  | F1 file | 250 | | 3.0 |
|  | F2 file | 250 | | 2.0 |


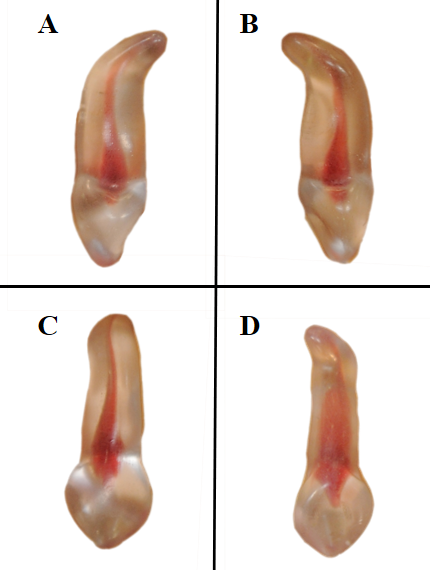


**Figure S1.** 3D printed resin teeth imitating the right maxillary canine. A: mesial surface, B: distal surface, C: labial surface, D: palatal surface.


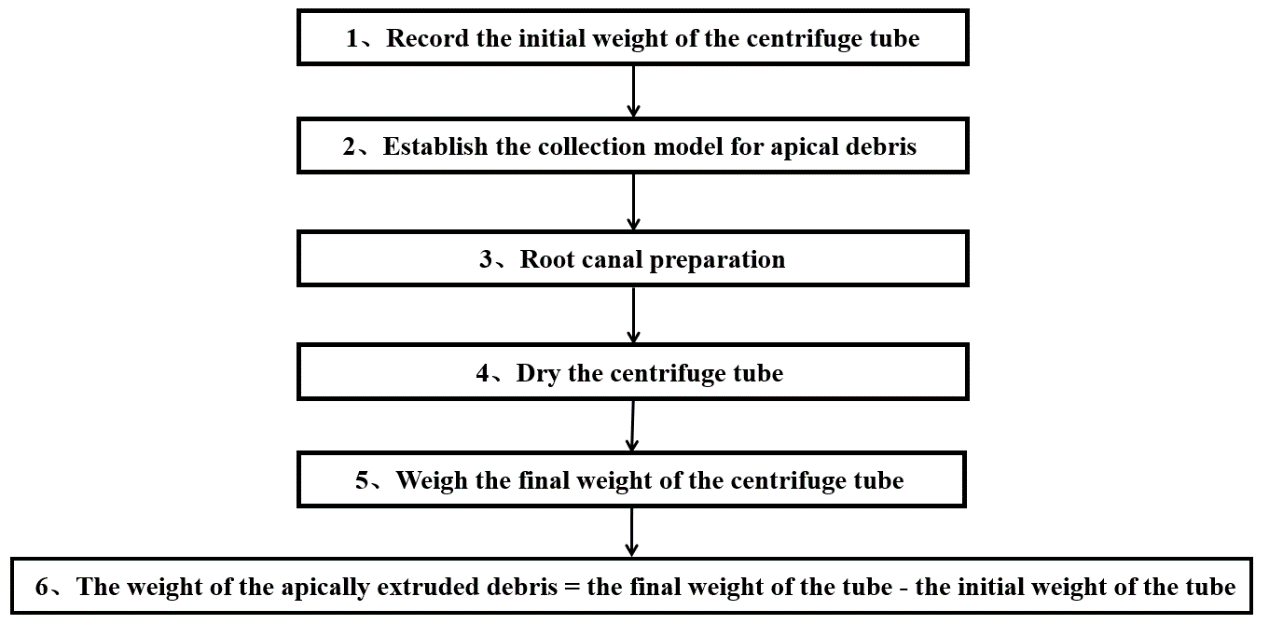


**Figure S2.** Schematic diagram for collection of apical debris.


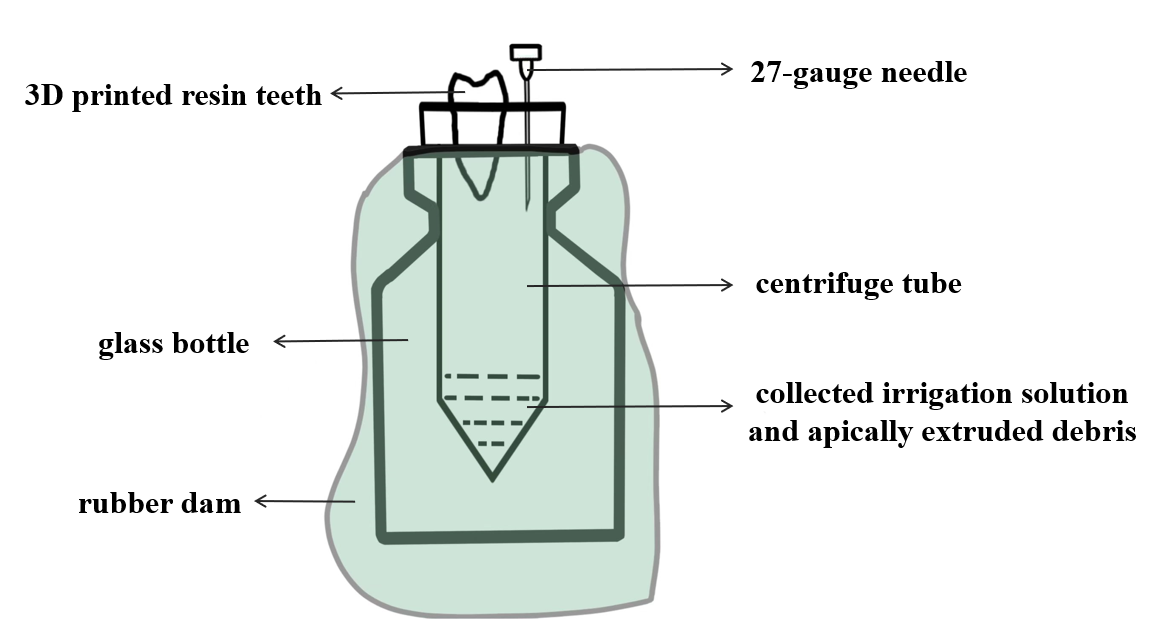


**Figure S3.** Schematic diagram of the apical debris collection model.


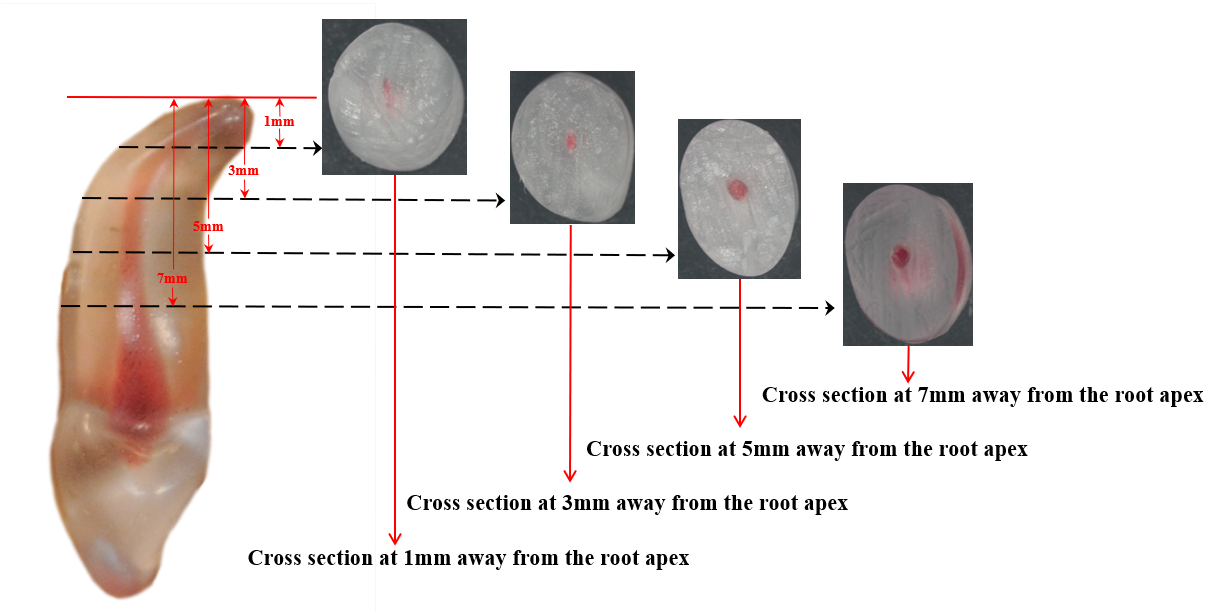


**Figure S4.** Schematic diagram of cross sections at different distance from the root apex.


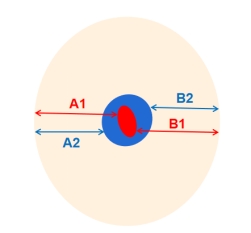


**Figure S5.** Schematic diagram showing the root canal transportation and centering ratio measurement. The red area represents the root canal lumen before root canal preparation, while the blue area represents the root canal lumen after root canal preparation.

**
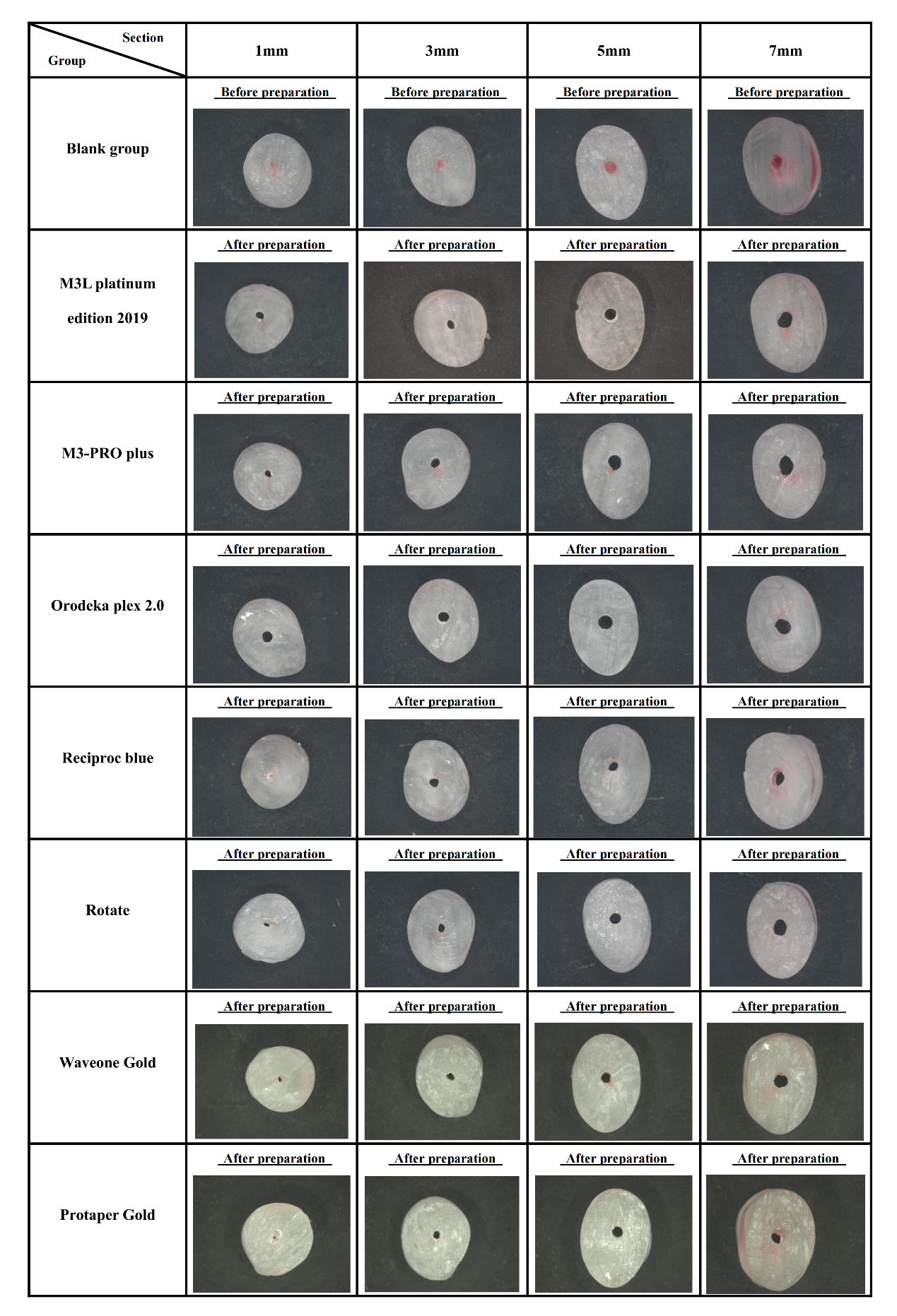
**

**Figure S6.** Cross-sectional images before and after root canal preparation in different experimental groups.
